# Toxicity of diatom-derived polyunsaturated aldehyde mixtures on sea urchin *Paracentrotus lividus* development

**DOI:** 10.1038/s41598-018-37546-y

**Published:** 2019-01-24

**Authors:** Nadia Ruocco, Concetta Annunziata, Adrianna Ianora, Giovanni Libralato, Loredana Manfra, Susan Costantini, Maria Costantini

**Affiliations:** 1 Department of Marine Biotechnology, Stazione Zoologica Anton Dohrn, Villa Comunale, 80121 Napoli, Italy; 2 Department of Biology, University of Naples Federico II, Complesso Universitario di Monte Sant’Angelo, Via Cinthia, 80126 Napoli, Italy; 3 Bio-Organic Chemistry Unit, Institute of Biomolecular Chemistry-CNR, Via Campi Flegrei 34, Pozzuoli, Naples 80078 Italy; 4 Institute for Environmental Protection and Research (ISPRA), Rome, Italy; 5 Cancer Research Center of Mercogliano, Istituto Nazionale Tumori –IRCCS - Fondazione G. Pascale, 80131 Napoli, Italy

## Abstract

Diatom-derived polyunsaturated aldehydes (PUAs), decadienal, heptadienal and octadienal, derive from the oxidation of fatty acids and have cytotoxic and anticancer effects. PUAs, tested separately, induce malformations in sea urchin *Paracentrotus lividus* embryos. Decadienal induces the worst malformations and lowest survival rates. Interestingly, decadienal, heptadienal and octadienal place in motion several genes to counteract their negative effects. To date, no studies are available reporting on the effects of PUA mixtures on marine invertebrates. Here we test binary and ternary mixtures on embryonic development of *P. lividus*. Our findings demonstrate that mixtures of PUAs act (i) at morphological level in synergistic way, being much more severe compared to individual PUAs; (ii) at molecular level also reveal an additive effect, affecting almost all fifty genes, previously tested using individual PUAs. This study is relevant from an ecological point of view since diatoms are a major food source for both pelagic and benthic organisms. This work opens new perspectives for understanding the molecular mechanisms that marine organisms use in reacting to environmental natural toxin mixtures such as diatom PUAs.

## Introduction

Marine organisms are constantly subjected to a mixture of environmental stressors and natural and/or dissolved anthropogenic compounds, including both physical and chemical^1^. In the last two decades several studies have been performed on chemical toxicants to address scientific questions in mixture toxicology^2–6^. Environmental policy has recognized in mixture effects the major issue in environmental risk assessment^2^. In a situation of exposure to multiple xenobiotics, single compounds may act independently as in a single exposure, or they may interact to modulate the effects of total multiple exposure^7^. The interactions of components in a mixture can result in synergistic, antagonistic or additive toxicity^8,9^.

Among natural toxins, representing a major source of stress for marine organisms, many studies have been performed on diatom-derived secondary metabolites known as oxylipins. These natural compounds are the end-products of a lipoxygenase/hydroperoxide lyase metabolic pathway, triggered by cell damage or breakage, as during grazing by predators or by lysis of cells at the end of diatom blooms^10–15^. Of the known oxylipins, PUAs are the most comprehensively studied because they were the first group described^16^ and are also commercially available, inexpensive and sufficiently stable to allow for a range of laboratory bioassays to be conducted. For these reasons, it has been extensively demonstrated that PUAs are able to affect the embryonic development of several marine invertebrates such as starfish and polychaetes^17–19^, ascidians^20,21^, copepods (reviewed by Ianora and Miralto)^22^ and sea urchins^23–31^. Moreover, in human cancer cell lines PUAs activated cell death^32^. All these studies have been performed testing the three most common PUAs, decadienal, heptadienal and octadienal, separately. At lower concentrations PUAs (decadienal from 0.5 to 2.5 μM, heptadienal from 1.0 to 6.0 μM; octadienal from 2.0 to 9.0 μM) induced a dose-dependent increase in the number of malformed embryos in the sea urchin *Paracentrotus lividus*, with decadienal inducing the strongest effects (2.0 μM)^29^. PUAs induced apoptosis in *P. lividus* embryos at higher concentrations (decadienal 3.3 μM, heptadienal 9.0 μM and octadienal 11.0 μM)^27^.

The potential level of real environmental exposure to the PUAs has been reported in Ribalet *et al*.^33^. These authors estimated that the release of PUAs from each diatom cell was 46.9, 4.7 and 0.5 μmol PUA l^−1^ considering a distance of 1, 10 and 100 μm from the cell surface. These data were well within the significant range for affecting growth and performance of surrounding organisms^22^.

In our studies, we used as model organism to test PUAs mixtures the sea urchin *P. lividus*, which is a well-established marine model species in eco-toxicological studies, being constantly exposed to environmental toxicants and emerging pollutants^34–36^ and very useful for experimental studies and microscopic observations thanks to its high fecundity and the large number of synchronous and transparent embryos produced^37,38^. In particular, we incubated *P. lividus* sea urchin eggs with binary and ternary mixtures of different PUA concentrations, fertilized and followed embryonic development until pluteus stages at 48 hours post fertilization. In order to identify genes affected by PUA mixtures, we also analyzed the expression levels of fifty genes (previously analyzed using the three PUAs separately), having a key role in a broad range of functional responses, such as development, differentiation, skeletogenesis, stress and detoxification processes. The rationale of this work is based on the consideration that organisms are exposed to oxylipin mixtures during direct feeding on diatoms or due to direct exposure, when these compounds are released in seawater following diatom cell lysis in the marine environment. Our aim was to study the possible toxigenic effects of PUA mixtures on sea urchin *P. lividus* embryos using morphological and molecular approaches.

## Results

### Effects of PUA mixtures on sea urchin development

To define PUA mixture effects on sea urchin embryos we checked the following endpoints in *P. lividus* reproduction and embryonic development: (i) fertilization success (immediately after the addition of sperm which induces an evident elevation of the fertilization membrane in sea urchin eggs); (ii) first cleavage division (leading to two blastomeres, at about 1 hpf); (iii) pluteus stage (at 48 hpf). The aldehyde mixtures tested refer to the concentrations reported in Varrella *et al*.^29^ which showed that concentrations of 1.6 µM decadienal, 3.0 µM heptadienal and 4.5 µM octadienal tested individually induced the same proportion of malformed embryos (about 35%). With the three binary PUA mixtures, (decadienal 1.6 µM + heptadienal 3.0 µM; decadienal 1.6 µM + octadienal 4.5 µM; heptadienal 3.0 µM + octadienal 4.5 µM) we observed 100% fertilization success, 100% first mitotic division after 1 hpf but after 48 hpf all embryos were apoptotic (Supplementary Fig. S1). With the ternary mixtures (decadienal 1.6 µM + heptadienal 3.0 µM + octadienal 4.5 µM) we also obtained 100% fertilization success, but only about 50% of fertilized eggs reached first cleavage and those that did had abnormal two blastomeres (Supplementary Fig. S2). As in the case of binary mixtures all embryos were apoptotic after 48 hpf.

In the second experiment, we halved the concentration of the three aldehydes (0.8 µM decadienal, 1.5 µM heptadienal and 2.3 µM octadienal). In this case the results were the same both for the binary and ternary PUA mixtures with regards to fertilization success (100%) first mitotic division (100%) and apoptotic embryos after 48 hpf (100%), with the only exception being the binary mixture heptadienal 1.5 µM + octadienal 2.3 µM. In this case the embryos observed at 48 hpf showed a developmental delay and were still at the early pluteus stage compared to control (without PUA mixture) embryos that had reached the late pluteus stage (Supplementary Fig. S3). We followed embryonic development of the early plutei until 1 week post fertilization (wpf) to follow their fate. These embryos reached the pluteus stage even if they were malformed (with degenerate and malformed arms, as pluteus in Supplementary Fig. S3a), that showed delayed development compared to controls (Supplementary Fig. S4), where the embryos showed the characteristic “ampoule-like” shape^31^.

**Figure 1:**
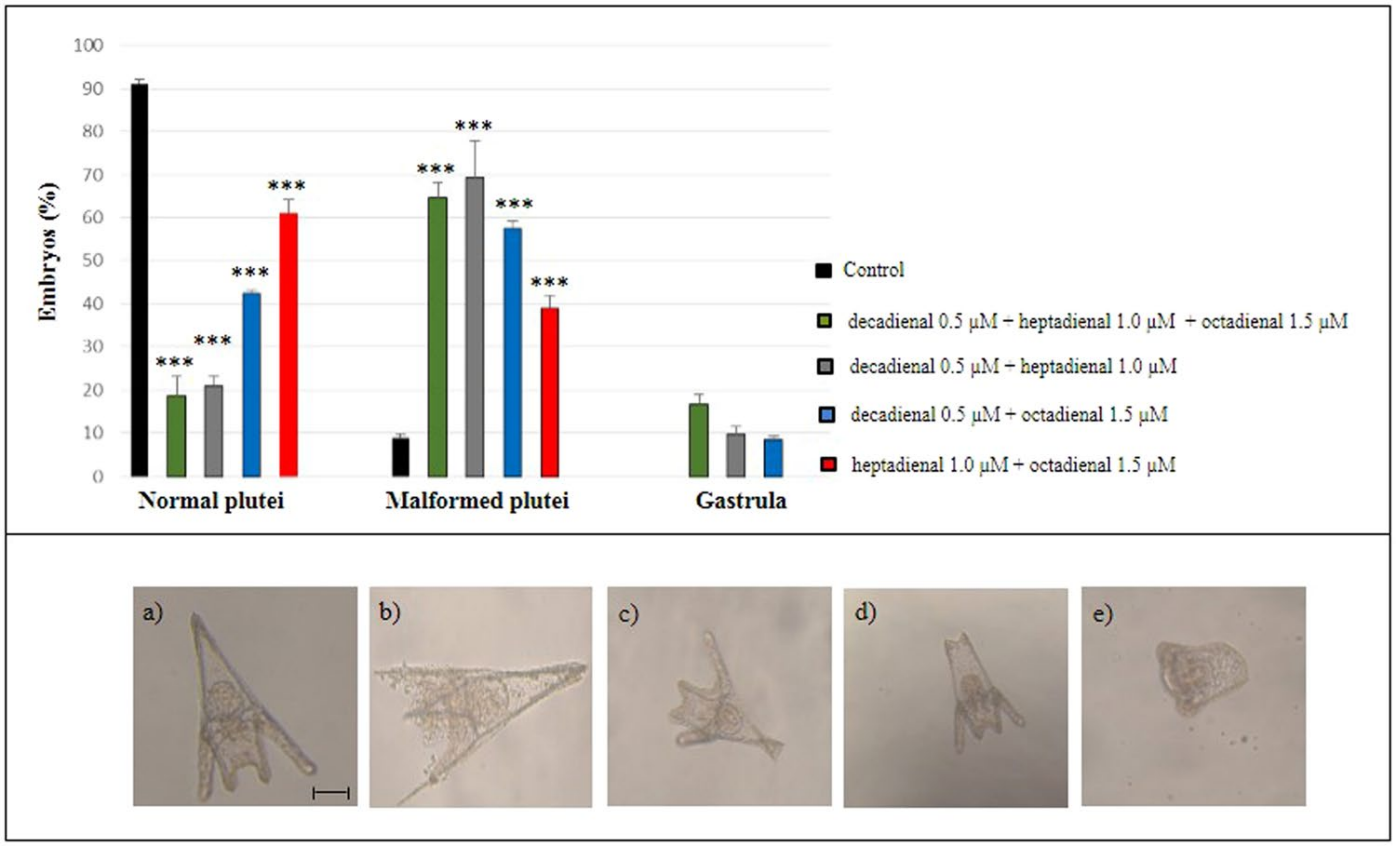
Examples of malformations induced in (**b**–**d**) *P. lividus* plutei at 48 hpf after incubation with binary (decadienal 0.5 µM + heptadienal 1.0 µM; decadienal 0.5 µM + octadienal 1.5 µM; heptadienal 1.0 µM + octadienal 1.5 µM) and ternary (decadienal 0.5 µM + heptadienal 1.0 µM + octadienal 1.5 µM) mixtures in comparison with (**a**) the control (embryos in sea water without PUA mixtures); (**e**) embryos still at the gastrula stage. Bar, 50 µm.

We then further decreased the three PUA concentrations in the mixtures to one third of the original concentrations, corresponding to 0.5 µM decadienal, 1.0 µM heptadienal and 1.5 µM octadienal. At these concentrations samples incubated with both binary and ternary mixtures showed 100% fertilization success, 100% division to two blastomeres at 1 hpf. At 48 hpf we observed a very high percentage of embryos at the pluteus stage in all the mixtures tested. More specifically, treatment of eggs with binary and ternary mixtures at 48 hpf produced a significant increase in the number of malformed embryos (p < 0.0001) with respect to the control (Fig. 1b–d). However, only with binary and ternary mixtures containing decadienal we also observed a low percentage of delayed embryos still at the gastrula stage (Fig. 1e).

### Gene response to PUA mixtures

**Figure 2:**
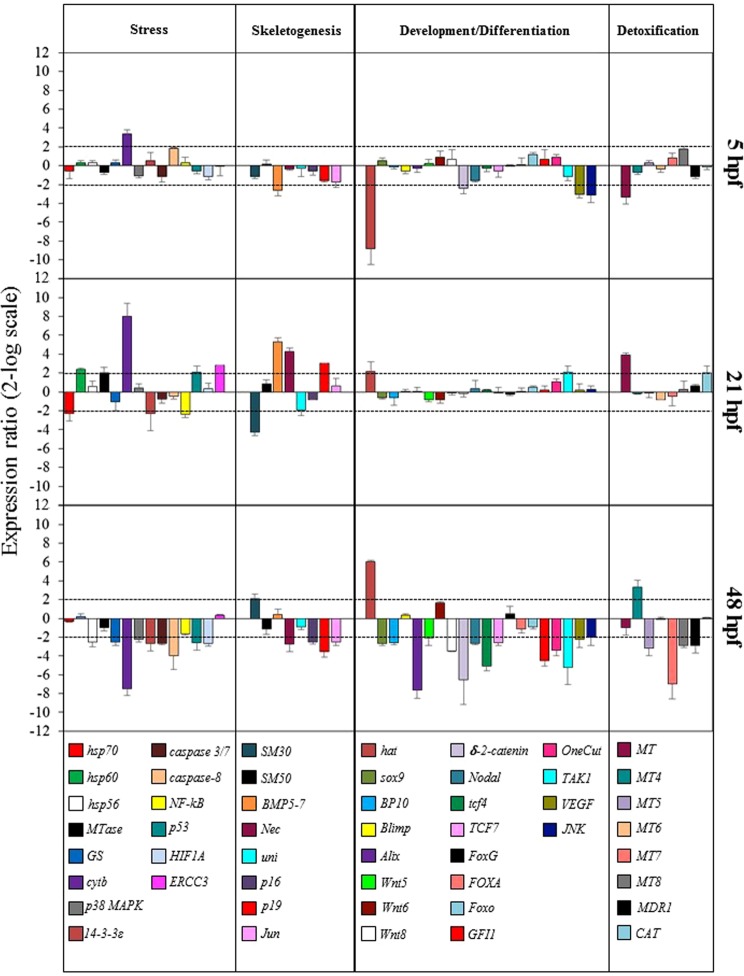
Real-Time qPCR at blastula (5hpf), gastrula (21 hpf) and pluteus (48 hpf) stages. Histograms show the differences in expression levels of fifty genes involved in different embryonic processes, divided in four classes: stress, skeletogenesis, development/differentiation and detoxification. *P. lividus* embryos were grown in the presence of ternary PUA mixture, consisting of decadienal 0.5 µM, heptadienal 1.0 µM and octadienal 1.5 µM. Data are reported as a fold difference compared with control (mean ± SD) embryos in sea water without PUA mixture. Fold differences greater than ±2 (see red dotted horizontal guidelines at values of +2 and −2) were considered significant (see Supplementary Table S2 for the values).

Sea urchin eggs were incubated with ternary mixtures of PUAs at concentrations that induced morphological changes but still allowed embryos to develop to the pluteus stage: 0.5 µM decadienal, 1.0 µM heptadienal and 1.5 µM octadienal. To identify the expression profile triggered by PUAs exposure, the expression levels of fifty genes were followed by Real Time qPCR, having key roles in embryonic development, cell differentiation and morphogenesis (see Supplementary Table S1). Their relative expression ratios were calculated respect to control embryos. We considered expression levels greater than two-fold with respect to the controls as significant (see Fig. 2 and Supplementary Table S2).

#### Stress genes

At the early blastula stage (5 hpf) the gene *cytb* were up-regulated (3.4-fold, p = 0.012) with respect to the control. At the gastrula stage (21 hpf) seven genes were targeted by the ternary PUA mixture: *hsp60* (2.4-, p = 0.048), *MTase* (2.0, p = 0.049), *p53* (2.1, p = 0.049) and *ERCC3* (2.8-, p = 0.035) were up-regulated, whereas *hsp70* (2.3-, p = 0.045), *14-3-3ε* (2.3-, p = 0.045) and *NF-kB* (2.4-, p = 0.041) were down-regulated with respect to the control. At the pluteus stage (48 hpf) nine genes were down-regulated, *hsp56* (2.5-, p = 0.045), *GS* (2.5-, p = 0.041), *cytb* (7.5, p = 0.0015), *p38MAPK* (2.3-, p = 0.039), 14-3-3ε (2.7-, p = 0.031), *caspase 3/7* (2.7-, p = 0.039), *caspase-8* (4.0-, p = 0.025), *p53* (2.6, p = 0.030) e *HIF1A* (2.7-, p = 0.031).

#### Skeletogenic genes

At the early blastula stage (5 hpf) only the gene *BMP5-7* was down-regulated (2.7-fold; p = 0.011) by the PUA mixture. At the gastrula stage (21 hpf) *SM30* and *uni* were down-regulated (4.3- and 2.0-fold with p = 0.019 and p = 0.042, respectively) whereas *BMP5-7*, *Nec* and *p19* were up-regulated (5.3-, 4.3- and 3.0-fold with p = 0.0011, 0.023 and 0.029, respectively). At the pluteus stage (48 hpf) SM30 was up-regulated (2.1-fold, p = 0.010), whereas *Nec*, *p16*, *p19* and *Jun* were down-regulated (2.8-, 2.5-, 3.6- and 2.5-fold with p = 0.011, p = 0.039, p = 0.0023, p = 0.027) with respect to the control.

#### Genes involved in development/differentiation

At the early blastula stage (5 hpf) all the targeted genes were down-regulated: *hat* (8.8-, p = 0.0054), *δ-2-catenin* (2.4-, p = 0.015), *VEGF* (3.1-, p = 0.010) and *JNK* (3.1-, p = 0.0012).

At the gastrula stage (21 hpf) *hat* and *TAK1*were down-regulated (2.2- and 2.1-fold, with p = 0.028 and p = 0.033 respectively). At the pluteus stage (48 hpf) hat gene was strongly up-regulated (6.0-fold, p = 0.0045). On the contrary, fourteen genes were down-regulated: *sox9* (2.7-, p = 0.019), *BP10* (2.6-, p = 0.034), *Alix* (7.6-, p < 0.0001), *Wnt5* (2.1-, p = 0.013), *Wnt8* (3.5-), *δ-2-catenin* (6.5-, p = 0.0028), *nodal* (2.7-, p = 0.021), *tcf4* (5.1-, p = 0.0017), *TCF7* (2.6-, p = 0.031), *GFI1* (4.5-, p = 0.0045), *Onecut*/*Hnf6* (3.4-, p = 0.01), *TAK1* (5.2-, p = 0.0025), *VEGF* (2.3-, p = 0.039) *JNK* (2.0-, p = 0.045).

#### Genes involved in detoxification

At the early blastula stage (5 hpf) only *MT* gene was down-regulated (3.4-fold, p = 0.018). At the gastrula stage (21 hpf) *MT* and *CAT* were up-regulated (3.9- and 2.0-fold with p = 0.016 and p = 0.043, respectively). At the pluteus stage (48 hpf) MT4 was up-regulated (3.3-fold, p = 0.021), whereas *MT5*, *MT7*, *MT8* e *MDR1* were down-regulated (3.2-, 7.0-, 2.9- and 2.9-fold with p = 0.011, p < 0.0009, p = 0.023, p = 0.019, respectively).

Statistically significant differences were found between gene expression values at 48 hpf versus both 5 hpf (p < 0.001) and 21 hpf (p < 0.0001).

Summarizing, PUA mixtures were able to switch on the expression levels of almost all analyzed genes, with the exception of *SM50*, *Blimp*, *Wnt6*, *FoxG*, *FOXA*, *Foxo* and *MT6* (for a synopsis see Supplementary Fig. S7). All gene functional classes were affected by PUA mixtures but at different developmental stages. At the blastula stage only seven genes were targeted by PUA mixtures in the four functional classes, probably since it is too early to detect an effect on gene expression at this stage. At the gastrula stage, the number of affected genes increased, with sixteen genes varying in their gene expression: stress genes (seven genes) and those involved in skeletogenesis (seven genes) were mainly affected, indicating that embryos started to react against these toxic aldehydes. At the pluteus stage, when the entire body was well-formed, thirty four genes were affected by the PUA mixture, demonstrating that the embryos were trying to defend themselves by placing in motion a greater number of genes. In particular, the PUA mixture mainly affected genes involved in stress (nine genes) and in development and differentiation processes (fifteen genes). It is important to underline that some previous results demonstrated that most of these genes were intercorrelated^28,30^.

### Heat maps of differentially expressed genes

We have constructed three heatmaps, one for each developmental stage, considering the three females for both control and after PUAs exposure (see Supplementary Fig. S5). Real-Time q PCR data were presented as individual data points as 2^−ΔC^_T_^39^. Our results showed that the three females in the control conditions (in seawater without PUAs, indicated with C1, C2 and C3) and after treatment with PUA mixture (indicated with T1, T2 and T3) clustered in two separate groups within the dendrogram. Moreover, differences within the same groups (within the control samples and treated samples) were also evident. These results reflected a fraction of the variability that is likely to be expected in this species.

**Figure 3:**
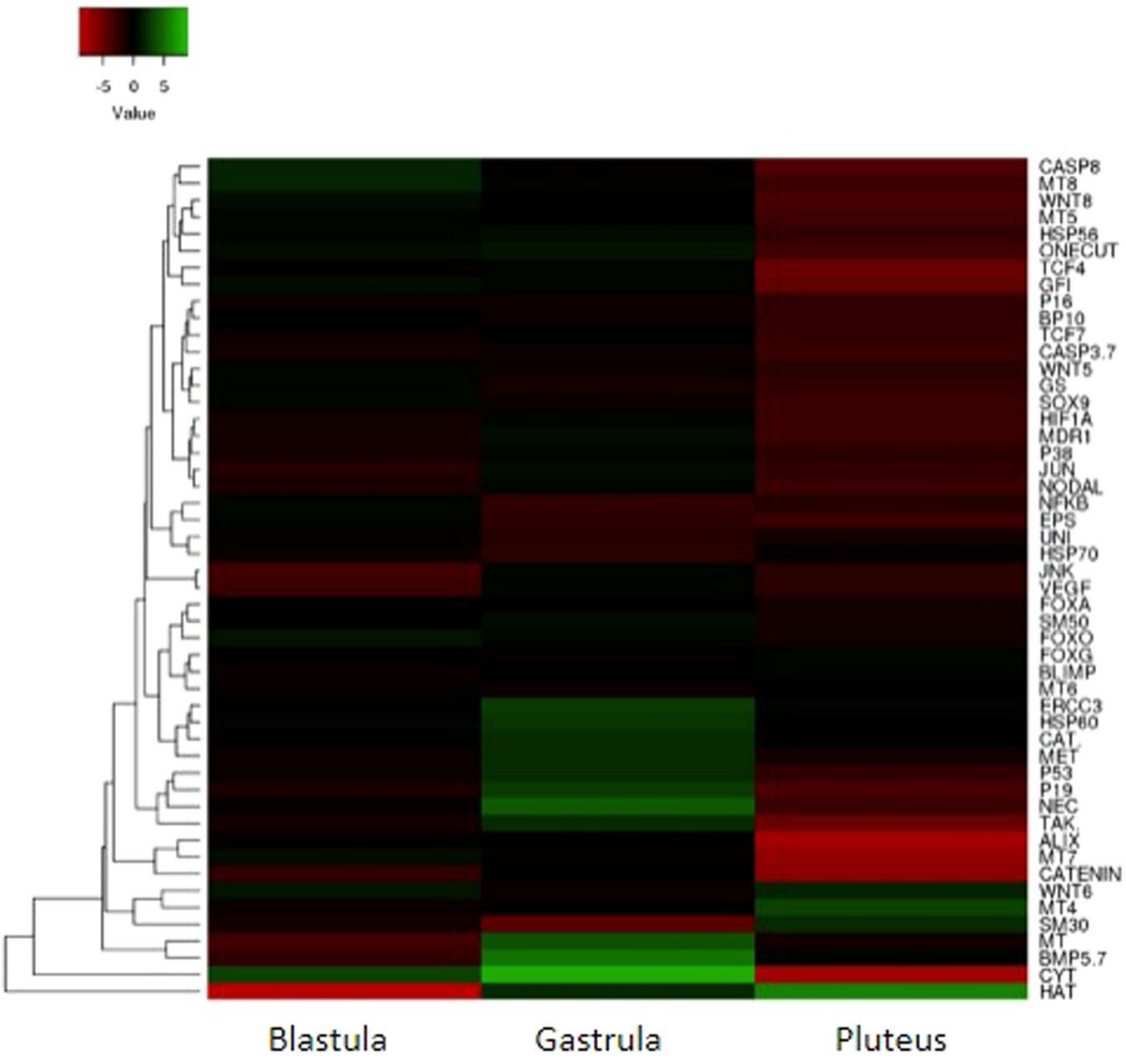
Heat map (using Heatmapper available at www.heatmappear.ca; https://creativecommons.org/licenses/by-sa/2.0/) of differentially expressed genes versus the three developmental stages (early blastula, late gastrula and pluteus), at which developing sea urchin *P. lividus* embryos have been collected after PUA mixture at 0.5 µM decadienal, 1.0 µM heptadienal and 1.5 µM octadienal for Real Time qPCR. Color code: red, negative values of gene expression (down-regulated genes respect to the control, embryos developed in sea water without PUA mixture); green, positive values of gene expression (up-regulated genes respect to the control); black, genes for which there was no variation of expression respect to the control.

A heat map of differentially expressed genes versus developmental stages is reported in Fig. 3, which shows the high level of gene expression variability among the three different developmental stages considered, increasing from the blastula stage to pluteus stage where a down-regulation of genes was very evident.

This high variability at the population level was also evident in the heat map of gene expression versus the three females (indicated with F1, F2 and F3; Supplementary Fig. S6), where F2 seems to differ somewhat in terms of its response in gene expression compared to F1 and F3.

## Discussion

In the present study, the effects of PUA mixtures on early stages of embryonic development in the sea urchin *P. lividus* are evaluated for the first time. These results greatly expand our previous investigations using single PUAs, decadienal, heptadienal and octadienal, to study the stress response induced of the sea urchin. Heptadienal and octadienal are two ecologically important diatom-derived PUAs, but relatively unknown in terms of their effects on grazers, in comparison with the extensively studied PUA, decadienal^24–30,40,41^. Our results also provide information on the transmission of “maternal” stress induced by PUAs to their progeny at the morphological and molecular levels. In sea urchin eggs maternal mRNAs drive the first mitotic cell cycles starting from fertilization to the onset of zygotic transcription, independently of mRNA transcription and ribosome biogenesis^42–44^.

Several ecotoxicological studies concern single compound exposures, although the toxicity of certain compounds increases in mixtures and in presence of other pollutants. Our findings take an alternative look at a relatively well-studied area of marine chemical ecology, making significant advances on PUA effects in mixtures and not limited to single PUA exposures.

To date, all the published data have reported the negative effects of individual PUAs on marine invertebrates. There are some studies reporting the effect of decadienal, as representative of PUAs, in the presence of sub-lethal levels of heavy metals on the rotifer *Brachionus plicatilis* and nauplii of the brine shrimp *Artemia salina*^45^. Also in this case, decadienal was the most toxic to both species. The presence of 1 μM of copper sulphate and zinc sulphate in solutions of decadienal resulted in an 11 and 3% reduction in the LD50 (Lethal Dose 50%, the concentration required to kill 50% of the test population) of decadienal compared to the 33% reduction with 1 μM copper sulphate.

Here, we investigated the effects induced not by individual PUAs, but by PUA mixtures. More specifically, we tested binary and ternary mixtures, demonstrating that PUAs in mixtures acted in a synergistic way, because their effect was stronger than individual PUAs, with negative impacts on sea urchin embryos. When we used binary and ternary mixtures at the concentrations that produced about 35% of malformed embryos with individual PUAs (decadienal 1.6 µM, heptadienal 3.0 µM and octadienal 4.5 µM)^29^, all the embryos were apoptotic. Only at PUA concentrations corresponding to one third of these concentrations (corresponding to decadienal 0.5 µM, heptadienal 1.0 µM and octadienal 1.5 µM), were embryos able to develop to the pluteus stage. According to Varrella *et al*.^29^ at these concentrations, individual PUAs have no effects on sea urchin embryos with values similar to the control (embryos in filtered sea water without PUAs). Moreover, our results confirm that decadienal was the strongest PUA, because all the PUA mixtures tested containing decadienal always had stronger toxicity for *P. lividus* embryos. These results corroborate previous data reporting that decadienal affected sea urchin embryonic development in a very narrow range (2.0 μM); whereas heptadienal and octadienal required higher ranges of concentrations to reach the same effects as decadienal^29^.

The stronger effects of PUA mixtures in comparison with individual PUAs observed at the morphological level, was also confirmed by molecular results. The expression levels of several genes in the sea urchin *P. lividus*, appeared to be modulated by individual PUAs decadienal, heptadienal and octadienal. These three aldehydes had some common targets (15 out of 50 genes analyzed), differently affecting the classes of genes at different embryonic stages^29^. In fact, heptadienal induces the strongest molecular effects, affecting the expression levels of thirteen genesbut at the pluteus stage^30^. Even if octadienal results the weakest of the three aldehydes by morphological point of view, our previous study on gene expression levels revealed that it affects early embryonic development. Decadienal seems to be molecularly the weakest of the three PUAs, but it has specific molecular targets. Therefore, although no morphologically differences are visible, PUAs affected different physiological processes.

Combining standard embryological assays with gene expression is useful to tease out interesting data in terms of how the PUA mixtures interact with embryo transcriptome. The present study highlights substantial gene plasticity in embryos of the sea urchin *P. lividus* in response to PUA mixtures. More specifically, embryos subjected to the PUA mixture reduced and delayed response at the transcript level, taking into account that the greatest number of genes was down-regulated and mainly at the pluteus stage. These data suggest that the physiological response in the sea urchin *P. lividus* may be impaired at the molecular level after PUAs mixture exposure. These data are in accordance with some experiments performed on the larvae from the red sea urchin *Strongylocentrotus franciscanus*, showing a reduced and delayed response in *hsp70* transcript level when exposed to elevated CO_2_ conditions and then subjected to a temperature shift^46^. In that case, the Authors suggested that marine organisms could protect themselves against ocean acidification, costing a lot for the organism’s ability to tolerate increased stress. Molecular plasticity has been previously demonstrated also in a mollusc population, when subjected to environmental stress^47,48^. In this case larger-scale genomic studies have been performed, appearing as one of the most rapid and sensitive way to study the responses to environmental stressors. Martin *et al*.^49^ focused their attention in understanding if ocean acidification affect the initial developmental stages of *P. lividus*. In fact, this organisms appeared extremely resistant to low pH, showing no effect on fertilization success or larval survival, but only delay of the larval development. The analyzed genes, involved in developmental and biomineralization processes, were surprisingly upregulated at low pH, so revealing that plasticity at the gene expression level was able to produce a normal, but delayed embryonic development under low pH conditions.

Finally, it would be interesting to consider PUA ratios, but to our knowledge, the only data available are those for *Skeletonema marinoi*, which does not produce decadienal but only heptadienal and octadienal. Vidoudez and Pohnert^50^ reported that heptadienal was at least 3 times more abundant than octadienal. Both PUAs were mainly produced in the late stationary phase when a dramatic increase in heptadienal and octadienal was observed, with concentrations reaching 290 nM heptadienal and 86 nM octadienal. These two PUAs were almost entirely absent in the other growth phases. There is also limited information for another diatom species, *Thalassiosira rotula*, which produces all three PUAs, but the only study available provides information on the total quantity of PUAs and not for each of the three individual aldehydes^51^.

## Conclusions

To our knowledges, this work represents the first report on the effects of diatom-derived PUAs mixtures on the sea urchins. Our findings reveal a synergistic toxic effect of diatom-derived aldehyde mixtures on embryonic *P. lividus* development, also confirming that the sea urchin embryos can be considered a good model to study the stress defence mechanisms in marine invertebrates. Finally, this represents an important ecological finding, considering that marine organisms are exposed to diatom-derived oxylipin mixtures, offering new knowledges on how marine organisms try to react and to defend themselves against environmental natural toxin mixtures such as diatom-derived PUAs.

## Materials and Methods

### Ethics Statement

*Paracentrotus lividus* (Lamarck) adults were collected in the Bay of Naples according to the Italian legislation (DPR 1639/68, 09/19/1980 confirmed on 01/10/2000). Field studies did not include endangered or protected species. All experimental procedures on animals were in compliance with the guidelines of the European Union (Directive 609/86).

### Morphological experiments

Concerning the collection of adult sea urchin *P. lividus* and the procedure for gametes collection see Varrella *et al*.^29,31^. Eggs were treated for 10 minutes with binary and ternary mixtures of 2-trans,4-trans-decadienal (Sigma-Aldrich), 2-trans,4-trans-heptadienal (Sigma-Aldrich), and 2-trans,4-trans-octadienal (Sigma-Aldrich) at the following concentrations:

1. 2*-trans*,4-*trans-*decadienal 1.6 µM, 2-*trans*,4-*trans-*heptadienal 3.0 µM, 2-*trans*,4-*trans*-octadienal a 4.5 µM;
2. 2*-trans*,4-*trans-*decadienal 0.8 µM, 2-*trans*,4-*trans-*heptadienal 1.5 µM, 2-*trans*,4-*trans*-octadienal 2.3 µM;
3. 2*-trans*,4-*trans-*decadienal 0.5 µM, 2-*trans*,4-*trans-*heptadienal 1.0 µM, 2-*trans*,4-*trans*-octadienal 1.5 µM;

Eggs were then fertilized. Experiments were conducted in triplicates for each group of concentrations, using eggs from ten different females. Control experiment consists in fertilizing eggs in filtered sea water without PUA mixtures. The development was followed until 48 hours post fertilization (hpf) and then embryos were fixed with formaldehyde (4% in FSW) in treated and control samples for microscopic observation, in order to detect the percentage of abnormal plutei, (Zeiss Axiovert 135TV, Carl Zeiss, Jena, Germany): control samples (in FSW without PUAs) showed about 90% of normal plutei and about 10% of malformed embryos.

Differences between normal and malformed plutei and embryos still at the gastrula stage exposed to different PUA mixtures were evaluated as mean ± standard deviation (SD) and Student’s *t* tests were performed with Prism 3.0 software (GraphPad Prism 4.00 for Windows, GraphPad Software, San Diego California USA). P < 0.05 was considered as statistically significant.

### Molecular experiments

Concerning the collection of eggs and total RNA extraction using RNAqueous® Micro Kit (Ambion from Life Technologies), see Ruocco *et al*.^52^. The expression level of 50 genes previously analyzed genes were followed by *Real-Time qPCR*^24–31^ (Supplementary Table S1). The expression of each gene was analyzed and internally normalized against *Pl-Z12-1* (a zinc-finger transcription factor)^53^ using REST software (Relative Expression Software Tool, Weihenstephan, Germany) based on the Pfaffl method^54,55^. PCR efficiencies were verified by melting curve analysis^24,28,30^. One microliter of cDNA was used as a template. For further details on quantification and qualitative analysis of RNA see Ruocco *et al*.^52^). Relative expression ratios above two cycles were considered significant. For each Real Time qPCR plate the experiments were repeated at least twice. We checked that all genes whose fold-change in expression was >±2 compared to the control were statistically significant. A Mantel test was applied.

Heat map of the differentially expressed genes versus developmental times for three different females were constructed using Heatmapper^56^ available at www.heatmappear.ca. A hierarchical clustering was performed through the Euclidean distance measurement method and an average linkage clustering method.

When real-time PCR data are presented as individual data points they should be presented as 2^−ΔC^_T_ or 2^−C^_T_ rather than the raw C_T_ value^39^. In this way, the data from gene expression profiling studies are normalize to an internal control. Heatmaps generally undergo a log transformation.

Concerning the morphological data to evidence differences between mixtures for both normal and malformed groups, statistical analysis was performed using GraphPad Prism version 4.00 for Windows (GraphPad Software, San Diego, CA, USA). Data of expression level did not pass the D’Agostino and Person normality test, indicating that One-way ANOVA was not applicable to the data sets. However, a non-parametric Kruskal-Wallis test was applied, followed by Dunn’s post-hoc test.

## Supplementary information

Supplementary Info
