# Supplementary material for: Toxicity of diatom-derived polyunsaturated aldehyde mixtures on sea urchin Paracentrotus lividus development
Source: Sci Rep. 2019 Jan 24;9:517. doi: 10.1038/s41598-018-37546-y (PMC6345956; doi:10.1038/s41598-018-37546-y)
Supplement: Supplementary file 1 — Supplementary Info [file 41598_2018_37546_MOESM1_ESM.docx]

**Toxicity of diatom-derived polyunsaturated aldehyde mixtures on sea urchin *Paracentrotus lividus* development**

**Nadia Ruocco^1,2,3,#^, Concetta Annunziata^1,#^, Adrianna Ianora^4^, Giovanni Libralato^2^, Loredana Manfra^1,5^, Susan Costantini^6^, Maria Costantini^1,^***

*^1^Department of Biology and Evolution of Marine Organisms, Stazione Zoologica Anton Dohrn, Villa Comunale, 80121 Napoli, Italy*

*^2^Department of Biology, University of Naples Federico II, Complesso Universitario di Monte Sant’Angelo, Via Cinthia, 80126, Napoli, Italy*

*^3^Bio-Organic Chemistry Unit, Institute of Biomolecular Chemistry-CNR, Via Campi Flegrei 34, Pozzuoli, Naples 80078, Italy*

*^4^Department of Integrative Marine Ecology, Stazione Zoologica Anton Dohrn, Villa Comunale, 80121 Napoli, Italy*

*^5^Institute for Environmental Protection and Research (ISPRA), Rome, Italy*

*^6^Unità di Farmacologia Sperimentale,, Istituto Nazionale Tumori - IRCCS - Fondazione G. Pascale, 80131 Napoli, Italy*

**^#^** These authors contributed equally to this work.

* Authors to whom correspondence should be addressed: maria.costantini@szn.it

Tel.: +39-081-583-3315; Fax: +39-081-764-1355

**Supplementary Fig. S1.** Photos (taken with Zeiss Axiovert 135TV microscope, 10x / 0.30, magnification / numerical aperture) of a) *P. lividus* embryos at the pluteus stage (at 48 hpf) in the control (embryos in sea water without binary PUA mixture) and b) embryos at 48 hpf in presence of binary PUA mixture (decadienal 1.6 µM + heptadienal 3.0 µM; decadienal 1.6 µM + octadienal 4.5 µM; heptadienal 3.0 µM + octadienal 4.5 µM). Bar, 50 µm.


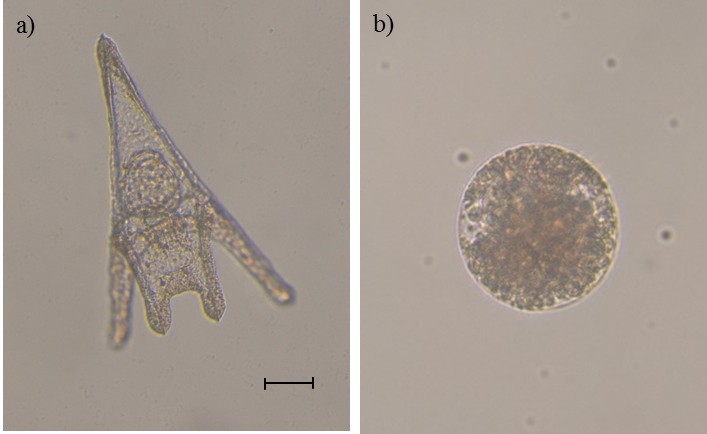


**Supplementary Fig. S2.** Photos (taken with Zeiss Axiovert 135TV microscope, 10x / 0.30, magnification / numerical aperture) of a) *P. lividus* fertilized eggs after the first cleavage division (about 1 hpf) at the two blastomeres stage in the control (embryos in sea water without binary PUAs mixture) and b-c) embryos at 1 hpf in presence of ternary PUA mixture (decadienal 1.6 µM + heptadienal 3.0 µM + octadienal 4.5 µM). Bar, 50 µm.


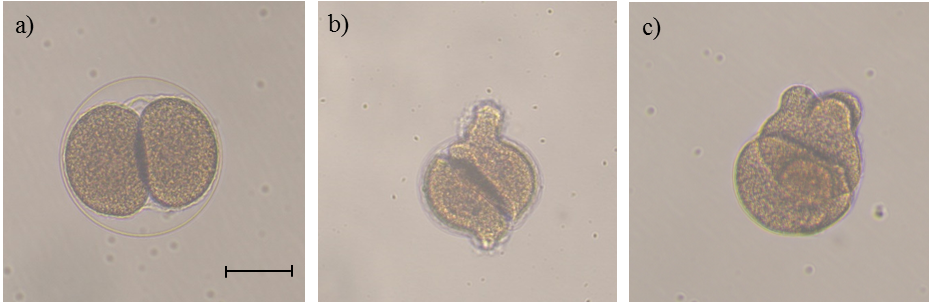


**Supplementary Fig. S3.** Photos (taken with Zeiss Axiovert 135TV microscope, 10x / 0.30, magnification / numerical aperture) of a) *P. lividus* embryos at the pluteus stage at 48 hpf in the control (embryos in sea water without binary PUAs mixture) and b) embryos at the early pluteus stage at 48 hpf in presence of binary PUA mixture (heptadienal 1.5 µM + octadienal 2.3 µM). Bar, 50 µm.


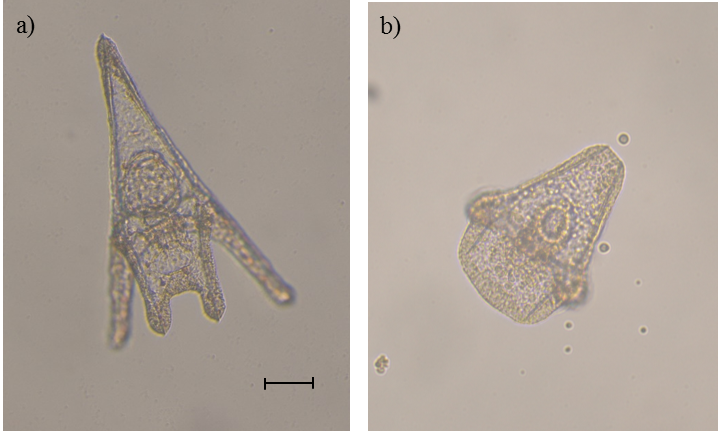


**Supplementary Fig. S4.** Photos (taken with Zeiss Axiovert 135TV microscope, 10x / 0.30, magnification / numerical aperture) of a) embryos at 1 wpf in the control (without binary PUA mixture) and b) embryos at 1 wpf grown in the presence of binary mixture heptadienal 1.5 µM + octadienal 2.3 µM. Bar, 50 µm.


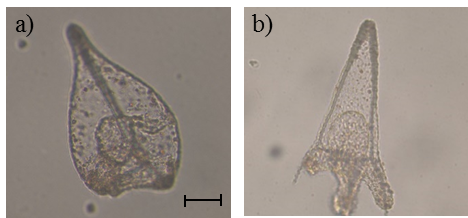


**Supplementary Figure S5.** Heat map of differentially expressed genes, one for each developmental stage, considering the three females for both control conditions (seawater without PUA mixture, indicated with C1, C2 and C3) and after PUAs exposure (indicated with T1, T2 and T3). Real-time qPCR data are presented as individual data points as 2^-ΔC^_T_. The data from gene expression profiling studies are normalize to an internal control. (Heatmapper available at www.heatmappear.ca).

**
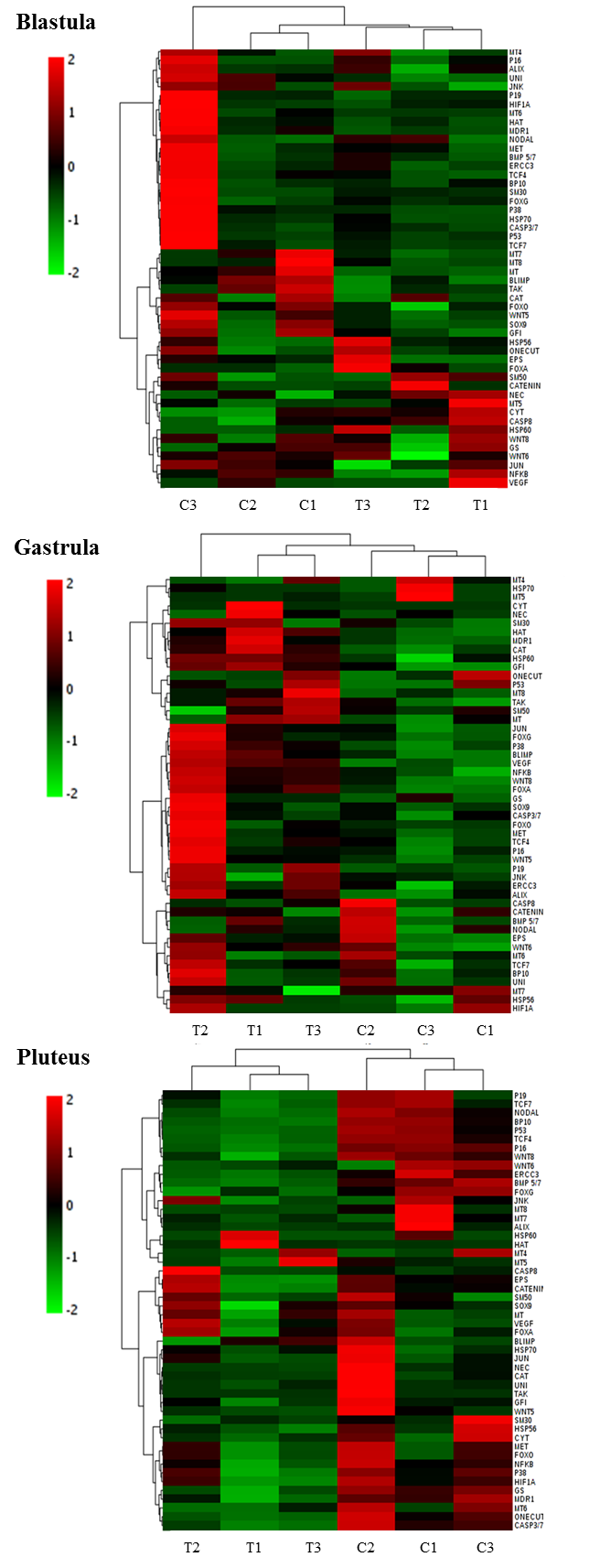
**

**Supplementary Figure S6.** Heat map of differentially expressed genes versus the three female (indicated with F1, F2 and F3) groups at three developmental stages, blastula, gastrula and pluteus (Heatmapper available at www.heatmappear.ca).

**
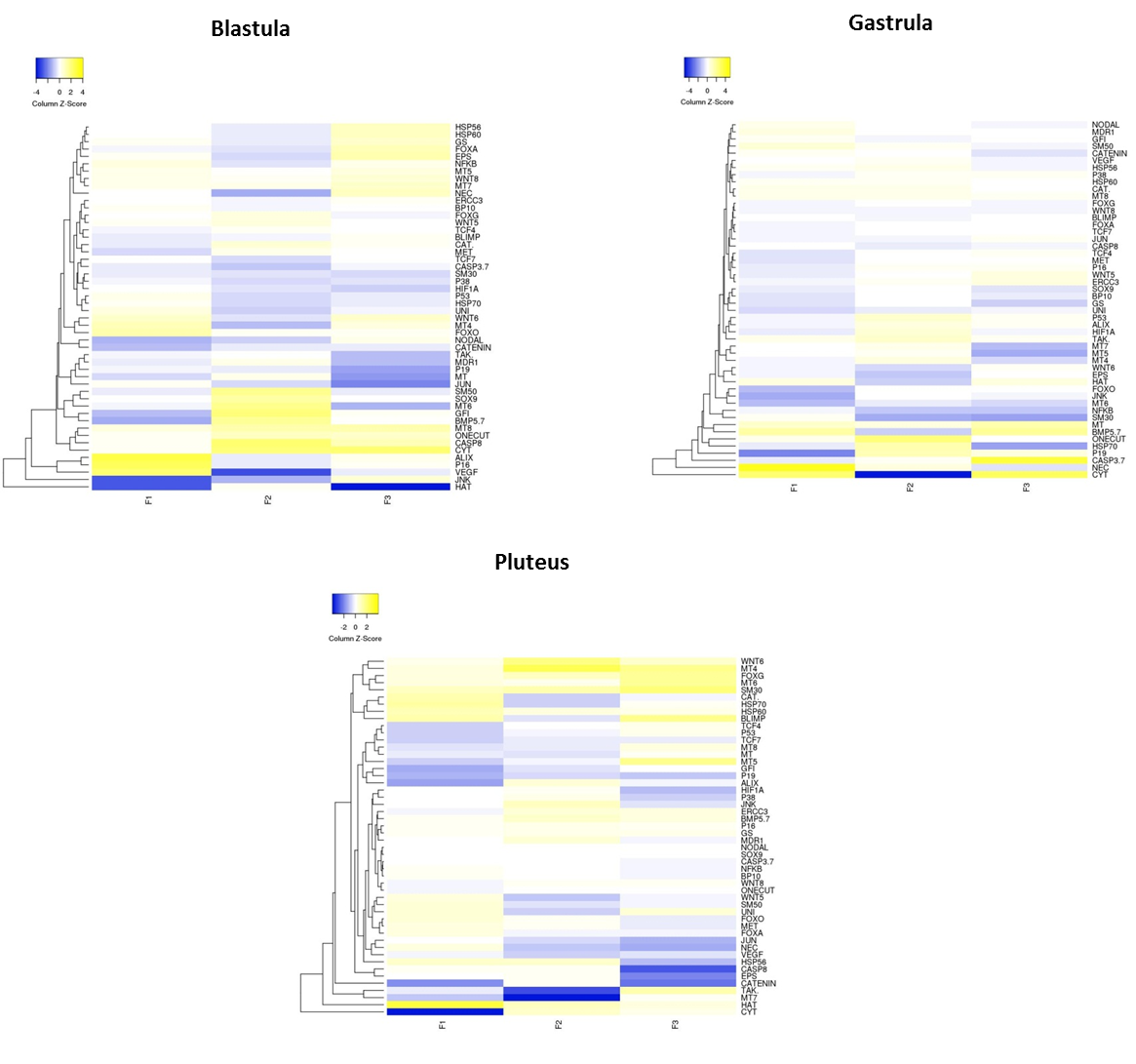
**

**Supplementary Figure S7.** Synopsis of the patterns of up- and downregulation of different classes of genes in the sea urchin *P. lividus* in the presence ternary PUA mixture, containing decadienal 0.5 µM, heptadienal 1.0 µM and octadienal 1.5 µM.


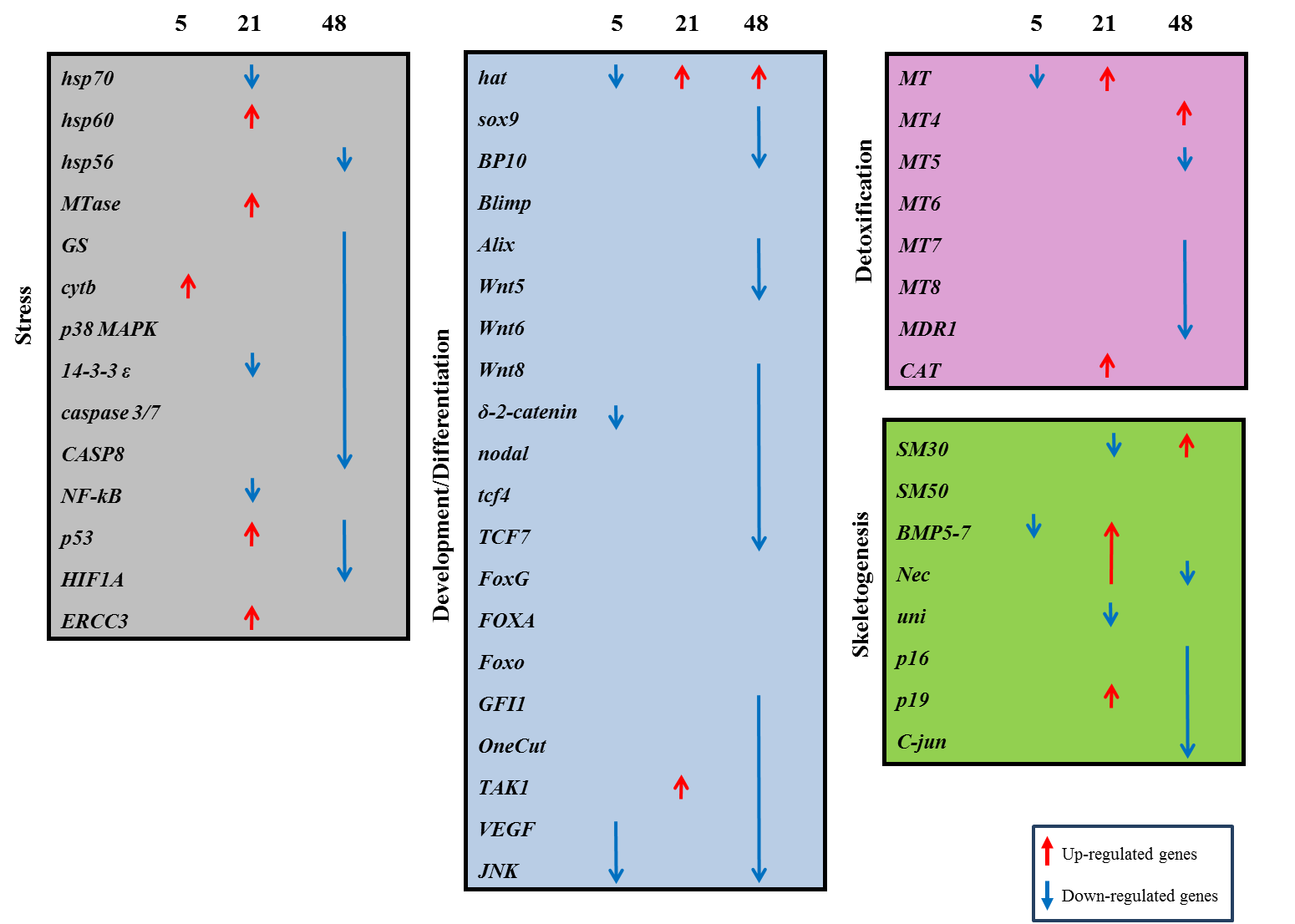


**Supplementary Table S1.** List of primers for the different genes, their sequences and length of PCR fragment in base pairs (bp).

|  |  |  |  |
| --- | --- | --- | --- |
| **Gene** | **Primer** | **Sequence 5'->3'** | **PCR fragment (bp)** |
|  |  |  |  |
| **Stress** |  |  |  |
| ***hsp70*** | Pl_hsp70_Up | CAGAACCACGCCCAGCTATG | 150 |
| (Romano et al., 2011) | Pl_hsp70_Rev | GCTTGGATGCTACTATCGTTG |  |
|  |  |  |  |
| ***hsp60*** | Pl_hsp60_F1 | GAATATCCAGTGTACTCCGAC | 160 |
| (Marrone et al., 2012) | Pl_hsp60_R1 | GCATCAGCTAAGAGGTCAAC |  |
|  |  |  |  |
| ***hsp56*** | Pl_hsp56_F1 | GGAGCTATGCTAAGGACATC | 183 |
| (Marrone et al., 2012) | Pl_hsp56_R1 | CTACAGCCTTAGCGACAGTG |  |
|  |  |  |  |
| ***MTase*** | Pl_Met1_F1 | GATCTCGTCAGACGATAGAAG | 175 |
| (Marrone et al., 2012) | Pl_Met1_R1 | CTCTTGCTGTGTTAGCATTG |  |
|  |  |  |  |
| ***GS*** | Pl_GS_F1 | GTGTCGGACCGATATCTGAC | 177 |
| (Marrone et al., 2012) | Pl_GS_R1 | CTCCGATTGATCCGTACTCG |  |
|  |  |  |  |
| ***Cytb*** | Pl_Cyt_F1 | GGGATACGTATTAGTCTGAGG | 144 |
| (Marrone et al., 2012) | Pl_Cyt_R1 | CGAGTTAGGGTGGCATTGTC |  |
|  |  |  |  |
| ***p38 MAPK*** | Pl_p38_F1 | GTGATCAGCTTGCTTGACTG | 144 |
| (Marrone et al., 2012) | Pl_p38_R1 | GTAGATGAGGAACTGGACGTG |  |
|  |  |  |  |
| ***14-3-3ε*** | Pl_Eps_F1 | CGGATAGATACAATGACATGG | 146 |
| (Marrone et al., 2012) | Pl_Eps_R1 | GCTGACTGTATGCAATGCTG |  |
|  |  |  |  |
| ***caspase-8*** | Cas8_Pl_F2 | GATACGACGAGCAGCGCAACATCTAG | 146 |
| (Romano et al., 2011) | Cas8_Pl_R2 | CTAGCATCATCCACTCTCATCCACTGCAC |  |
|  |  |  |  |
| ***caspase 3/7*** | Sp_Cas7_F2L | CTTCTTTATACAGGCATGCAGAGGCACCCAGATG | 175 |
| (Ruocco et al., 2016) | Sp_Cas7_R2L | CCGCCACGAGTAGTAACCTGGCGTTGATGAAAATGC |  |
|  |  |  |  |
| ***NF-κB*** | Pl-NF-kB_F | TCCCATGGAGGACTGCCGTGTCA | 116 |
| (Varrella et al., 2016) | Pl-NF-kB_R | TCGTTGGTTACCAAGGAGACCACA |  |
|  |  |  |  |
| ***p53*** | Sp_p53_F1 | GCGTTGGTGGATCATACTGG | 163 |
| (Varrella et al., 2016) | Sp_p53_R1 | GATCTTGGTCTGAGCGTAGTG |  |
|  |  |  |  |
| ***HIF1A*** | Sp_HIF1A_F1 | CGATAGAAGAGATCATCGACTC | 158 |
| (Varrella et al., 2016) | Sp_HIF1A_R1 | GTAGTCGTAGATGCTCTGGC |  |
|  |  |  |  |
| ***ERCC3*** | Pl_XPB_ERCC3_F | CAGGTTTCATCCCATGGTGGATCA |  |
| (Russo et al., 2014) | Pl_XPB_ERCC3_R | ATACTCCTCCGCTGCAGCACCT |  |
|  |  |  |  |
| **Skeletogenesis** |  |  |  |
| ***SM30*** | Pl_SM30_F1 | TTGGGTTCAGTTGGAGAACC | 384 |
| (Marrone et al., 2012) | Pl_SM30_R1 | GTTTCGTTGTCTTCGGGGTA |  |
|  |  |  |  |
| ***SM50*** | Pl_Sm50_F1 | GATGGCACACCAGCTTATCC | 162 |
| (Marrone et al., 2012) | Pl_Sm50_R1 | CTGACGCTTCATGACTGGAG |  |
|  |  |  |  |
| ***BMP5-7*** | Pl_BMP_F1 | TGGCAGGAATGGATCATCGC | 117 |
| (Marrone et al., 2012) | Pl_BMP_R1 | GAGTGTCTGCACGATGGCGTG |  |
|  |  |  |  |
| ***Nec*** | Pl_Nec_F1 | CAAGCACAGCTGGGAATGG | 158 |
| (Marrone et al., 2012) | Pl_Nec_R1 | GGTCATTTGTTCTTGCACTC |  |
|  |  |  |  |
| ***uni*** | Pl_Uni_F1 | ACTGGATCATCGCTCCGATG | 259 |
| (Marrone et al., 2012) | Pl_Uni_R1 | CATCGGCATCCACAAGCTTC |  |
|  |  |  |  |
| ***p19*** | Pl_P19_F1 | GACAAGCTCGACATCAACAAG | 205 |
| (Varrella et al., 2014) | Pl_P19_R1 | CTGGAGTCGATGCTGCATCATG |  |
|  |  |  |  |
| ***p16*** | Pl-p16 For | CGGGCAGCGATGACTCA | 104 |
| (Varrella et al., 2014) | Pl-p16 Rev | AAATGCCATACCGCTCTTCTGT |  |
|  |  |  |  |
| ***Jun*** | Pl_JUN_F1 | GAGACTCAGTTCTACGAAGATTCAC | 139 |
| (Ruocco et al., 2017) | Pl_JUN_R1 | GCAAGCTTGAGCATCTGTACGT |  |
|  |  |  |  |
| ***hat*** | Pl_Hat_F1 | CCACTGTGCATAATGTCCCG | 138 |
| (Marrone et al., 2012) | Pl_Hat_R1 | CAGAGACAGGAGTCAGTAGAC |  |
|  |  |  |  |
| **Development/Differentiation** | |  |  |
| ***sox9*** | Pl_Sox9_F1 | GAGCTTCATCACTCCCTGTC | 175 |
| (Marrone et al., 2012) | Pl_Sox9_R1 | GATGGATGGAGAGAACTGCG |  |
|  |  |  |  |
| ***BP10*** | Pl_Bp10_F1 | CTACGGGTGATCAGAAGGAG | 156 |
| (Marrone et al., 2012) | Pl_Bp10_R1 | CTTCAGTGAGCATCATGTCTC |  |
|  |  |  |  |
| ***Blimp*** | Pl_Blimp1_For | CTGTCTACTCCATGCCGTCC | 161 |
| (Varrella et al., 2014) | Pl_Blimp1_Rev | GCCTCCTGCTTCAGATCAGC |  |
|  |  |  |  |
| ***Alix*** | AL1500for | TACCAGACCATTCTCAACAAT | 110 |
| (Varrella et al., 2014) | AL1610rev | TGCTATTTCCGCTTCGCTTTT |  |
|  |  |  |  |
| ***Wnt 5*** | Pl_Wnt5_F2 | CACCCAGCCCCTGTGCAGTG | 135 |
| (Varrella et al., 2014) | Pl_Wnt5_Rev | CTGCAGTTCCACCTCCTATTC |  |
|  |  |  |  |
| ***Wnt 6*** | Pl_Wnt6_For | CGAATCTGCCGACGATCACG | 164 |
| (Varrella et al., 2014) | Pl_Wnt6_For | GCATTGTCGTACAGTTCCACC |  |
|  |  |  |  |
| ***Wnt 8*** | Pl_Wnt8_For | CTGTAAGTGTCATGGCGTCTC | 197 |
| (Varrella et al., 2014) | Pl_Wnt8_For | GAGCGAATCGGAGATGACGG |  |
|  |  |  |  |
| ***δ2-catenin*** | Sp_Catenin_F1 | GGATACTCAATCAAGATCACAAC | 229 |
| (Varrella et al., 2016) | Sp_Catenin_R1 | CTCTGACAGTACAATGAGATATGG |  |
|  |  |  |  |
| ***nodal*** | Pl_Nodal_F1 | CAACTCACGGATCATCTCTC | 197 |
| (Ruocco et al., 2017) | Pl_Nodal_R1 | CGATGGTGAGGAAGCAGACG |  |
|  |  |  |  |
| ***tcf 4*** | Pl_TCF4_F2 | GATGATGACCAGTCCGATACCAG | 177 |
| (Ruocco et al., 2017) | Pl_TCF4_R2 | GAGCGCTACAGGATGAATAC |  |
|  |  |  |  |
| ***TCF7*** | Sp_TCF7_F2 | GTCTAGTTTGATCGACGAGGGA | 153 |
| (Ruocco et al., 2017) | Sp_TCF7_R2 | GCAATGTGGTCGAGTTTGGAC |  |
|  |  |  |  |
| ***FoxG*** | Pl_FoxG_F1 | GACACACGCCTCCTCATCATCA | 160 |
| (Ruocco et al., 2017) | Pl_FoxG_R1 | CATCTTCCTCGTCGCTCTTG |  |
|  |  |  |  |
|  |  |  |  |
| ***FOXA*** | Pl_FOXA_F4 | GCGCCACGCTACATAAGATG | 150 |
| (Ruocco et al., 2017) | Pl_FOXA_R4 | GATACTGGCTGGATGCATTC |  |
|  |  |  |  |
| ***Foxo*** | Pl_Foxo_F1 | GGCACGACAGCCACCTGGA | 119 |
| (Ruocco et al., 2017) | Pl_Foxo_R1 | CAGGGTCGTTGTCAACCATTG |  |
|  |  |  |  |
| ***GFI_1*** | Pl_GFI1_F1 | CCCACGCGCGTCATCAACAC | 182 |
| (Ruocco et al., 2017) | Pl_GFI1_R1 | GTTAACCTCGTCGACCTCCG |  |
|  |  |  |  |
| ***OneCut/Hnf6*** | Pl_OneCut/Hnf6_F2 | GATCTCTAACACCAAAGTCT | 179 |
| (Ruocco et al., 2017) | Pl_OneCut/Hnf6_R2 | CACTTGCTCTTCCACGTTAGC |  |
|  |  |  |  |
| ***TAK1*** | Pl_TAK1_F2 | CCTGCGTGTTGCAAACTGAA | 181 |
| (Ruocco et al., 2017) | Pl_TAK1_R2 | GTCGAGCTGAGACCTTCTTCG |  |
|  |  |  |  |
| ***VEGF*** | Pl_VEGF_F1 | CACCCGAAGCCAGGCCCATG | 151 |
| (Ruocco et al., 2017) | Pl_VEGF_R1 | GCATGGGTGAGAAGTGGTTG |  |
|  |  |  |  |
| ***JNK*** | Pl_JNK_F2 | GATCGCTGATCTTCTCGGGAC |  |
| (Ruocco et al., 2017) | Pl_JNK_R2 | CTGGCTTGTGCACACATAG |  |
|  |  |  |  |
|  |  |  |  |
| **Detoxification** |  |  |  |
| ***MT*** | Pl_Mt_F1 | GAAAGCAGTGTCCCTGTGCAG | 162 |
| (Marrone et al., 2012) | Pl_Mt_R1 | CATGTACAGTTCCCTTCAGTG |  |
|  |  |  |  |
| ***MT4*** | MT4 For | GCTCAAAATCTTCAACATGGCTAATGA |  |
| (Varrella et al., 2014) | MT4 Rev | AGCACTTTCCAGTTTCACAACAAGC |  |
|  |  |  |  |
| ***MT5*** | MT5 For | CGACTTTAGCTCAAATTCATCACCATG |  |
| (Varrella et al., 2014) | MT5 Rev | TCCACAGCATTTACCATCCTTGC |  |
|  |  |  |  |
| ***MT6*** | MT6 For | CACGATTTGTGCTCAATCCTTCAT |  |
| (Varrella et al., 2014) | MT6 Rev | TTTGTGCATGATGTTCCACAGC |  |
|  |  |  |  |
| ***MT7*** | MT7 For | CGTCAAGAGATCAAAATCATCAACCA |  |
| (Varrella et al., 2014) | MT7 Rev | ACAGCACTCGCCAGTAATACAGCAC |  |
|  |  |  |  |
| ***MT8*** | MT8 For | GATGGTTGTCGTCGCTCCTAACA |  |
| (Varrella et al., 2014) | MT8 Rev | TCAAGAAAGGCTGGTATCAAATCTGAC |  |
|  |  |  |  |
| ***MDR1*** | Pl_MDR1_F2 | GTCAAGGTACTCAATGGGGTC | 158 |
| (Varrella et al., 2014) | Pl_MDR1_Rev | CGGATGTCAATGCCATCAATC |  |
|  |  |  |  |
| ***CAT*** | Sp-CAT_F1 | GACTTCGTCTTCACCGACGAG | 156 |
| (Varrella et al., 2014) | Sp-CAT_R1 | GACTCAAAGGGTGCAGCCTTG |  |

**Supplementary Table S2.** Data of expression levels were reported as a fold difference from control at 5 (blastula stage), 21 (gastrula stage) and 48 (pluteus stage) hpf after treatment with ternary PUA mixture, consisting of decadienal 0.5 µM, heptadienal 1.0 µM and octadienal 1.5 µM. (See also Figure 2). Fold differences greater than ± 2 were considered significant. Significant p-values have also been reported.

|  | **5 hpf** | **p-value** | **21 hpf** | **p-value** | **48 hpf** | **p-value** |
| --- | --- | --- | --- | --- | --- | --- |
| Stress |  |  |  |  |  |  |
| *hsp70* | -0.6 |  | -2.3 | 0.045 | -0.3 |  |
| *hsp60* | 0.3 |  | 2.4 | 0.048 | 0.2 |  |
| *hsp56* | 0.3 |  | 0.6 |  | -2.5 | 0.045 |
| *Mtase* | -0.7 |  | 2.0 | 0.049 | -1.0 |  |
| *GS* | 0.3 |  | -1.1 |  | -2.5 | 0.041 |
| *cytb* | 3.4 | 0.012 | 8.0 |  | -7.5 | 0.002 |
| *p38 MAPK* | -1.1 |  | 0.4 |  | -2.3 | 0.039 |
| *14-3-3*ɛ | 0.5 |  | -2.3 | 0.045 | -2.7 | 0.031 |
| *caspase 3/7* | -1.2 |  | -0.8 |  | -2.7 | 0.039 |
| *caspase-8* | 1.8 |  | -0.4 |  | -4.0 | 0.025 |
| *NF-kB* | 0.3 |  | -2.4 | 0.041 | -1.7 |  |
| *p53* | -0.6 |  | 2.1 | 0.049 | -2.6 | 0.030 |
| *HIF1A* | -1.1 |  | 0.3 |  | -2.7 | 0.031 |
| *ERCC3* | -0.1 |  | 2.8 | 0.035 | 0.3 |  |
|  |  |  |  |  |  |  |
| Skeletogenesis |  |  |  |  |  |  |
| *SM30* | -1.1 |  | -4.3 | 0.019 | 2.1 | 0.0100 |
| *SM50* | 0.2 |  | 0.8 |  | -1.2 |  |
| *BMP5/7* | -2.7 | 0.011 | 5.3 | 0.001 | 0.4 |  |
| *Nec* | -0.4 |  | 4.3 | 0.023 | -2.8 | 0.0110 |
| *uni* | -0.3 |  | -2.0 | 0.042 | -0.9 |  |
| *p16* | -0.6 |  | -0.8 |  | -2.5 | 0.0390 |
| *p19* | -1.6 |  | 3.0 | 0.029 | -3.6 | 0.0023 |
| *Jun* | -1.8 |  | 0.7 |  | -2.5 | 0.0270 |
|  |  |  |  |  |  |  |
| Development/Differentiation |  |  |  |  |  |  |
| *hat* | -8.8 | 0.005 | 2.2 | 0.028 | 6.0 | 0.0045 |
| *sox9* | 0.5 |  | -0.6 |  | -2.7 | 0.0190 |
| *BP10* | -0.1 |  | -0.6 |  | -2.6 | 0.0340 |
| *Blimp* | -0.6 |  | 0.0 |  | 0.3 |  |
| *Alix* | -0.3 |  | 0.0 |  | -7.6 | <0.0001 |
| *Wnt5* | 0.2 |  | -0.9 |  | -2.1 | 0.0130 |
| *Wnt6* | 0.9 |  | -0.8 |  | 1.7 |  |
| *Wnt8* | 0.7 |  | -0.1 |  | -3.5 | 0.0014 |
| δ*-2-catenin* | -2.4 | 0.015 | -0.2 |  | -6.5 | 0.0028 |
| *Nodal* | -1.6 |  | 0.4 |  | -2.7 | 0.0210 |
| *tcf4* | -0.3 |  | 0.2 |  | -5.1 | 0.0017 |
| *TCF7* | -0.6 |  | 0.0 |  | -2.6 | 0.0310 |
| *FoxG* | 0.0 |  | -0.2 |  | 0.5 |  |
| *FoxA* | 0.1 |  | 0.1 |  | -1.1 |  |
| *Foxo* | 1.2 |  | 0.4 |  | -0.9 |  |
| *GFI1* | 0.7 |  | 0.2 |  | -4.5 | 0.0045 |
| *Onecut* | 0.9 |  | 1.1 |  | -3.4 | 0.0010 |
| *TAK1* | -1.2 |  | 2.1 | 0.033 | -5.2 | 0.0025 |
| *VEGF* | -3.1 | 0.010 | 0.2 |  | -2.3 | 0.0390 |
| *JNK* | -3.1 | 0.001 | 0.3 |  | -2.0 | 0.0450 |
|  |  |  |  |  |  |  |
| Detoxification |  |  |  |  |  |  |
| *MT* | -3.4 | 0.002 | 3.9 | 0.016 | -1.0 |  |
| *MT4* | -0.7 |  | -0.2 |  | 3.3 | 0.021 |
| *MT5* | 0.3 |  | -0.1 |  | -3.2 | 0.001 |
| *MT6* | -0.4 |  | -0.8 |  | 0.0 |  |
| *MT7* | 0.8 |  | -0.5 |  | -7.0 | <0.0009 |
| *MT8* | 1.7 |  | 0.2 |  | -2.9 | 0.002 |
| *MDR1* | -1.1 |  | 0.6 |  | -2.9 | 0.019 |
| *CAT* | -0.2 |  | 2.0 | 0.043 | 0.1 |  |
